# A Method for Prediction of Femoral Component of Hip Prosthesis Durability due to Aseptic Loosening by Using Coffin/Manson Fatigue Model

**DOI:** 10.1155/2018/9263134

**Published:** 2018-05-23

**Authors:** Branislav Krivokapic, Zoran Blagojevic, Dora Selesi, Teodor Atanackovic, Stevan Pilipovic, Zoran Bascarevic, Vladan Stevanovic

**Affiliations:** ^1^Institute for Orthopedic Surgery and Traumatology “Banjica”, Mihajla Avramovica 28, 11000 Belgrade, Serbia; ^2^Medical School, University of Belgrade, Serbia; ^3^Department of Mathematics and Informatics, Faculty of Sciences, University of Novi Sad, Trg D. Obradovica 4, 21000 Novi Sad, Serbia; ^4^Department of Mechanics, Faculty of Technical Sciences, University of Novi Sad, Trg D. Obradovica 6, 21000 Novi Sad, Serbia

## Abstract

The purpose of this work is to develop a new model estimate of the fatigue life of a hip prosthesis due to aseptic loosening as a multifactorial phenomenon. The formula developed here is a three-parameter model based on Basquin's law for fatigue, eccentric compression formula for the compressive stress and torsion in the prosthesis due to the horizontal components of the contact force. With our model, we can accurately predict the durability of a hip prosthesis due to the following four parameters: body weight, femoral offset, duration, and intensity of daily physical activities of a patient. The agreement of the prediction with the real life of the prosthesis, observed on 15 patients, is found to be adequate. Based on the formula derived for a particular implant, there was a high degree of concurrence between the model-predicted and actual values of aseptic loosening (durability) proved by the Mann–Whitney *U* test. By virtue of the validated model, it is possible to predict, quantitatively, the influence of various factors on the hip life. For example, we can conclude that a 10% decrease of a patient's body mass, with all other conditions being the same, causes 5% increase of the hip fatigue life.

## 1. Introduction

### 1.1. Purpose of the Modeling

In this paper, we aim to develop a theoretical modeling approach to predict prosthesis longevity prior to failure from aseptic loosening depending on both patient and implant data. It must be emphasized that this is not a statistical approach and therefore it does not require a large sample size. Established on prominent bioengineering and mechanical results such as the Coffin-Manson model [1] and Basquin's law for material fatigue [2, 3], we develop our model and thereafter utilize the data from three patients to create the coefficients for the model. These coefficients depend on the implant's material and therefore will always be prosthesis specific. The model we have developed can be recreated and replicated by any different group of researchers based on the data of only 3 patients with the same implant type following the same procedure as we did. We have used nonparametric statistical methods on 15 other patients to evaluate the agreement between the model predictions and recorded values, but not to develop the model itself. After creating the model with all coefficients, the model's prediction formula relies on several patient specific input parameters such as the patient's body weight *Q*, daily physical activity time *T*, femoral offset *h*, and a certain weight factor *K* that incorporates all effects due to patients' variable lifestyle habits depending on their type of physical stress loading [4].

While statistical prediction models such as generalized regression, survival analysis, or machine learning models such as bootstrapping or random forests require large sample sizes with minimum 20 patients per variable included, our model exhibits modesty since it requires data of only three patients due to its theoretical mathematical nature. Additionally, the subsequent statistical analysis we have conducted indicates a high degree of concurrence between the model-predicted and actual values of time to aseptic loosening proving the effectiveness of the model.

### 1.2. Clinical State of the Arts

Hip osteoarthritis is one of the most common degenerative diseases in adults. Prior to the age of 50 about 5% of people have degenerative joint disease involving the hip; however, between the ages of 50 and 65 the prevalence increases to 25% and after the age of 70, the risk jumps to 70% [5]. Total hip arthroplasty (THA) is currently one of the most widely performed procedures in orthopedic practice in the world, with approximately 800,000 to 1,000,000 operations per year [6, 7]. The outcomes of total hip arthroplasty are, in general, favorable and lead to a majority of satisfied patients [8]. Indeed, more than 90% of patients achieve almost complete pain relief and significant improvement in function [9]. There are different methods that can be used to assess the fatigue life for a hip prosthesis [10]. Moreover, in the last twenty years new methods, new approaches, and experimental and statistical analysis extend the level of the hip replacement theory and practice.

Aseptic loosening is a multifactorial phenomenon that is either due to initial lack of osteointegration (short term) or due to later failure of the bone-stem interface (long term). All this is caused by the concurrence of a number of factors including bone quality, surgical preparation of the host bone, type of stem surface, presence of wear debris, loading and lifestyle, and patients' age. Here we study the long term aseptic loosening, due to the patients' weight, lifestyle, and femoral offset. Having in mind all those factors, our aim in this study was to develop a mathematical model which could use patient specific data to predict the fatigue life of the bone-stem interface that leads to aseptic loosening. The Coffin/Manson formula based on the Basquin law was proposed in [3] to be a predictive measure of fatigue in structures and this fact is the cornerstone of our model.

## 2. Methods

### 2.1. Selection of Patients and Materials

We retrospectively reviewed data of 18 patients (12 female and 6 male) who underwent total hip arthroplasty in the year 1990 and who subsequently went on to revision surgery. All patients were operated on by one of two experienced reconstructive surgeons through a posterolateral approach. In all cases, the implant that was used was non-cemented hydroxyapatite (HA) coated femoral stem (RCM; Cremascoli Ortho, Milan, Italy). The average age of patients in this study group was 38.2 years (range, 35–55 years). Inclusion criteria for choosing patients were accurate size and positioning of femoral stem and young and active patients with good bone quality (we saw it during revision surgery) and exclusion criteria were significant dysplasia of the hip, patients with systemic diseases, and patients with significant preoperative functional disability.

**Figure 1:**
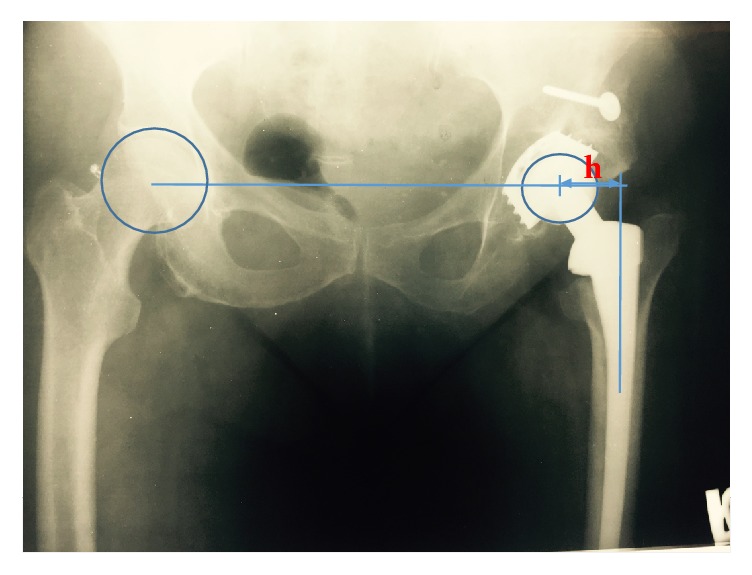
AP X-ray of the pelvis.

The femoral offset (*h*) was measured between the center of the head of the hip prosthesis and the imaginary line through the center of the femoral component in all patients on a standard anterior-posterior radiograph of the pelvis (see Figure 1). Note that *h* is determined by the reference length of the head component of the prosthesis for which we know the exact size and the corresponding ratio that we measured on the anterior-posterior radiograph of the pelvis.

Excessive physical activity of a patient results in loosening of the total hip arthroplasty (see [11], p. 891). The active lifestyle and daily habits of our patients, together with their weight, were registered in the medical records during the yearly routine outpatient clinics. We have prepared a Questionnaire (in the Appendix) based on the EuroQol 5D (EQ-5D) which is a standardized and validated questionnaire [6] but modified it to focus only on questions related to the patient's type and excess of physical activities. The Questionnaire consists of three groups of questions related to daily activities (household activities, sports activities, and job-related activities) scored on a five-point ordinal score scale and on a continuous visual scale. Patients were asked to fill out the Questionnaire on a yearly basis at each follow-up visit to the orthopedist's office and final scores were obtained by rating the combined responses to questions and averaging them out through all the yearly follow-ups. These scores were turned to normalized scores (Table 1) ranging from 0 to 1, with 0 denoting low physical activities, 0.5 moderate (average) physical activities, and 1 excessive activities.

### 2.2. Model Development

The fatigue life of a periodically loaded structure may be determined from the Wöhler curve in a stress-controlled experiment—see Meyers and Chawla [3]. On the basis of such an analysis, the so-called strain-life approach was adapted and applied in many biomechanical problems. In our analysis, we will take the stress-life curve according to Basquin's law in the form [12], p. 898

(1) $$\begin{array}{c} S_{amp}=\sigma_{f}^{\prime }{\left(\;N\;\right)}^{c}, \end{array}$$

where *S*_amp_ is the stress amplitude, *σ*_*f*_′ is the stress ductility coefficient,* c* is the stress ductility exponent, and *N* is the number of cycles before failure. In some published texts instead of *N* the authors use the number of reversals *N*_rev_ so that in formulas like (1) instead of *N* they use 2*N*_rev_ [13]. There are several variants of strain (or stress) fatigue life approach leading to the Coffin-Manson equation [1]. In some approaches both elastic and plastic strains are taken into account [13]. When elastic strain amplitude is taken into account, Basquin's law takes on the form of the Coffin-Manson equation; that is, (1) becomes

(2) $$\begin{array}{c} \frac{\Delta \varepsilon_{el}}{2}=\frac{\sigma_{f}^{\prime }}{E}{\left(\;N\;\right)}^{b}, \end{array}$$

(see [13] p. 716, [14]), where Δ*ε*_el_ denotes the elastic strain amplitude, *N* denotes the number of cycles before failure, *E* is the modulus of elasticity of the material, and *σ*_*f*_′ and *b* are constants. In (2) we did not take into account the creep strain rate *dε*_el_/*dt*. It was shown in [15] that this is really the case for a normal human bone. The power law dependence of the lifetime and external load amplitude, such as (1), exhibits universal features [2]. The exponent *b* has strong material dependence and is determined from experimental data. When the amplitude of the plastic strain is used in (2), the Coffin-Manson equation (see [3], p. 718) becomes

(3) $$\begin{array}{c} \frac{\Delta \varepsilon_{pl}}{2}=\varepsilon_{f}^{\prime }{\left(\;N\;\right)}^{c}, \end{array}$$

where *ε*_*f*_′ is the plastic strain ductility coefficient and *c* is the plastic strain ductility exponent. Note that there are various combinations of (2) and (3) such as (see [12])

(4) $$\begin{array}{c} \frac{\Delta \varepsilon }{2}=\frac{\sigma_{f}^{\prime }}{E}{\left(\;N\;\right)}^{b}+\varepsilon_{f}^{\prime }{\left(\;N\;\right)}^{c}, \end{array}$$

where Δ*ε* is the (total) strain amplitude. Basquin's law in its form (1) was successfully used in predicting fatigue life of endodontic instruments in [14]. It is a special form of (4) and in the sequel we shall use (1) and concentrate on the determination of *S*_amp_ that has a specific form for hip prosthesis.

In order to determine *S*_amp_, we note that the contact stress at hip-femur connection has two components: normal and shear. We treat first the normal stress as a combination of compression and bending; that is,

(5) $$\begin{array}{c} \sigma =\sigma_{comp}+\sigma_{bend}, \end{array}$$

where *σ*_comp_ is the stress due to compression and *σ*_bend_ is the stress due to bending. Following [16], from Figure 1, we conclude that the normal stress *σ* on the hip-femur connection is given as

(6) $$\begin{array}{c} \sigma =\frac{F}{A}\left(\;1+\frac{h}{{i_{x}}^{2}}y_{max}\;\right), \end{array}$$

where *F* is the weight of the patient acting on the hip, *A* is the cross-sectional area of the hip implant at the point of contact with the femur, *i*_*x*_^2^ is the radius of gyration with respect to the principal axes of the hip implant, *y*_max_ is the maximal distance from the principal axis of the hip to a point on the boundary of the hip implant, and *h* is the distance denoted on Figure 1. The stress *σ* given by (6) is transmitted to the femur. The force *F* in (6) acting on the hip is assumed to be the vertical component of the total force. We determine the effective force of the patient, determined from the weight of the patient and a weight coefficient *k*_1_. Thus, we assume that

(7) $$\begin{array}{c} F=k_{1}W=k_{1}\left(\;Qg\;\right), \end{array}$$

where *W* is the weight of the patient, *k*_1_ is a weight coefficient, *Q* is the mass of the patient, and *g* is the acceleration of the gravity. From [17], we cite the values of *k*_1_ as

(i) normal walking 3.5 km/h
  (8) $$\begin{array}{c} k_{1}=2.2, \end{array}$$
(ii) sudden stop while keeping balance
  (9) $$\begin{array}{c} k_{1}=3, \end{array}$$
(iii) stumbling without falling
  (10) $$\begin{array}{c} k_{1}=7.2. \end{array}$$

Recently in [18] the value of *k*_1_ is presented for physically demanding occupational tasks. Note that an experiment whose results are presented in [19] uses *k*_1_ = 1.4.

**Figure 2:**
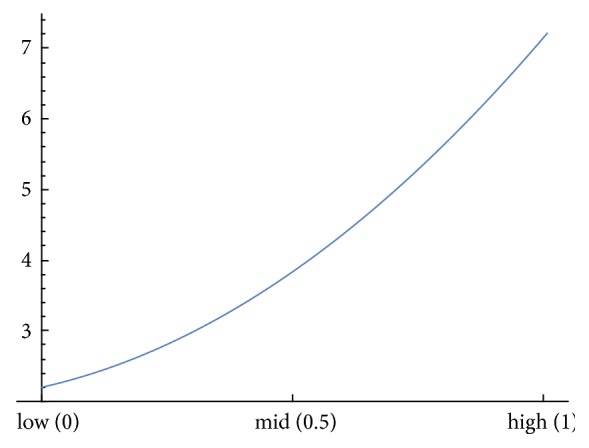
Cubic spline interpolation for *k*_1_.

In our analysis, the value of *k*_1_ for each particular patient is chosen on the basis of his/her physical activity and lifestyle. In order to determine approximate values of *k*_1_ for each patient, we adopted a cubic spline interpolation between the values *k*_1_ = 2.2 for low physical activities (normalized score equal to 0), *k*_1_ = 3 for moderate physical activities (normalized score equal to 0.5), and *k*_1_ = 7.2 for excessive physical activities (normalized score equal to 1). The computations were carried out in Wolfram Mathematica software (Figure 2). For each patient, we calculated their corresponding value of *k*_1_ (Table 1) based on this interpolation formula and on their scores obtained from the Questionnaire. Thus, for example, patient number 17 who was involved in high-impact activities like playing tennis and running during all years got a normalized score of physical excess activities equal to 0.975 resulting in *k*_1_ = 6.99, while patient number 7 who had a sitting job with only household activities scored only 0.414 resulting in *k*_1_ = 0.414.

Concerning the dynamic load during walking, as a function of time, we refer to [11]. Recall from [20] that the force action on a hip has three components: vertical (*F*_*z*_) and two in a horizontal plane (*F*_*x*_ and *F*_*y*_). In [20], it was shown that the vertical component *F*_*z*_ is the largest and that the other two may be neglected. However, *F*_*y*_ causes much of the implant torque. This torque may be represented as (see [20] p. 868)

(11) $$\begin{array}{c} M_{tr}=k_{2}W, \end{array}$$

where *k*_2_ is characteristic for the patient activity and *W* is the body weight of the patient. The shear stress coming from *M*_tr_ is given as

(12) $$\begin{array}{c} \tau =\frac{M_{tr}}{I_{0}}R=k_{2}\frac{W}{I_{0}}R, \end{array}$$

where *I*_0_ is the polar moment of inertia and *R* is the radius of the prosthesis ad the contact point with femur. Since *I*_0_ = 2*I*_*x*_ = 2*Ai*_*x*_^2^ and *i*_*x*_ = *R* the total equivalent normal stress (the principal stress, see [16] p. 30) that we use in (1) is

(13) $$\begin{array}{c} \sigma_{eq}=\frac{\sigma }{2}+\frac{1}{2}\sqrt{\sigma^{2}+4\tau^{2}}. \end{array}$$

We assume that the implant suffers fatigue when the crack occurs in the hip-femur connection.

By using (6), (7), and (13) in (1), we get (*y*_max_ = *R*, since the cross-section of the prosthesis at the point of contact is circular)

(14) $$\begin{array}{c} \frac{KQg}{A}\left(\;1+\frac{h}{i_{x}^{2}}y_{max}\;\right)=\sigma_{f}^{\prime }{\left(\;N\;\right)}^{c}, \end{array}$$

where

(15) $$\begin{array}{c} K=\frac{1}{2}k_{1}\left[\;1+\sqrt{1+4{\left[\frac{k_{2}R}{k_{1}i_{x}^{2}\left(1+\left(h/i_{x}^{2}\right)y_{max}\right)}\right]}^{2}}\;\right] \end{array}$$

is the modified load coefficient that takes into account torsion. Note that, for the case when torsion is neglected, we obtain *K* = *k*_1_. In general *k*_1_ and *k*_2_ are given in [20] for different patient activities. The values for normal walking are (see [20] p. 868) *k*_1_ = 2.38, *k*_2_ = 0.013 meters. Equation (14) may be written as

(16) $$\begin{array}{c} QK\left(\;1+c_{1}h\;\right)=c_{2}{\left(\;N\;\right)}^{c}, \end{array}$$

where *c*_1_ = *y*_max_/*i*_*x*_^2^ and *c*_2_ = *σ*_*f*_′*A*/*g*. The constants *c*, *c*_1_, and *c*_2_ in (15) are parameters of the model that are unknown. The number of cycles before failure *N* is determined as follows. We assume that we know the fatigue life of prosthesis in months *N*_*M*_. We take from [11] that a normal step takes about *T*_step_ = 1.11 seconds. Let *T* be the number of walking hours per day of a patient. Then the number of steps before the failure is

(17) $$\begin{array}{c} N=30N_{M}T\times \frac{3600}{T_{step}}, \end{array}$$

where *T*_step_ is the time of a single step in seconds. We take the value *T*_step_ = 1.11 as suggested in [11] p. 49. Thus, if we know *Q*, *K*, *h*, *T*, and *N*_*M*_ for three patients, we can determine *c*, *c*_1_, and *c*_2_. Let *Q*_*i*_, *K*_*i*_, *h*_*i*_, *T*_*i*_, and *N*_*M*_*i*__, *i* = 1,2, 3, be the values of body mass of a patient, weight coefficient, the length *h*_*i*_ specific for each patient, *T*_*i*_ number of walking hours per day, and life of the hip prosthesis in months, respectively, for three patients. We substitute this in (14) to obtain three equations of the type $Q_{i}K_{i}\;\;\left(1+c_{1}h_{i}\right)={\overset{-}{c}}_{2}{\left(T_{i}N_{M_{i}}\right)}^{c}$, where the constant ${\overset{-}{c}}_{2}$ is given as ${\overset{-}{c}}_{2}=c_{2}{\left(30\times 3600/T_{step}\right)}^{c}$. By the use of standard procedures, for example, Wolfram Mathematica software, we can solve the equations for the constants *c*, *c*_1_, and ${\overset{-}{c}}_{2}$. Note that *c*_1_ is expressed in (millimeter)^−1^.

In the analysis that follows, we choose the value of coefficient *K* since we do not have enough information on the *k*_2_/*k*_1_ ratio. This is equivalent to *k*_2_ = 0 so that *K* = *k*_1_. It is obvious that further study of influence of shear must be done. Since the number of patients in our study is rather small we could not present it here. Here we did this calculation and obtained the coefficients in (16) so that our final equation taken in the form (16) with *TN*_*M*_ instead of *N* reads

(18) QK1+0.0555886592×h=2.61607405×109TNM−2.187401.

Recall that *Q* is expressed in kilograms, *h* in millimeters (see Figure 1), *T* in hours per day, and *N*_*M*_ in months. The formula provided in (17) is our central result. The value for *K* can be estimated from (15) and the scores can be obtained from the Questionnaire in the Appendix. Since the coefficients are based on the preliminary data obtained from patients who underwent THA using the non-cemented RCM prosthesis and because the formula is prosthesis specific, the above formula can only be applied to the same prostheses. New coefficients can be obtained for any prosthesis and the coefficients will likely be subtly different from prosthesis to prosthesis.

## 3. Results

Based on Section 2.1 and the calculation of parameters by the data of three patients, formula (18) was used to calculate the predicted durability of each person's prosthesis for 15 patients to assess regarding the compliance between predicted and actual results.

### 3.1. Statistical Validation of the Model

**Figure 3:**
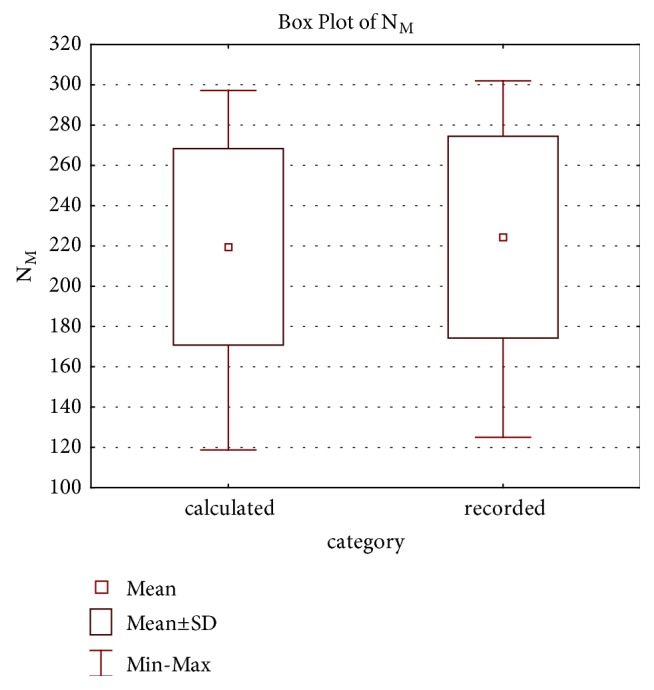
Comparison of the recorded values of* N* and the calculated values by model (18).

Calculated values of *N*_*M*_ ranged from 118.7 to 297.2 with a mean value of 219.55 and standard deviation of 48.74, while recorded values ranged from 125 to 302 with a mean value of 224.33 and standard deviation of 50.08. The Mann–Whitney *U* test of ranks was performed to compare the distribution of calculated values and recorded values of the prosthesis duration, resulting in acceptance of the hypothesis of their equality (*p* = 0.713). This indicates that there is a high concurrence between the two data sets and therefore proves the model provided by (18) as a valid one (Figure 3). Statistical analysis was performed in software Statistica (by Statsoft, Dell).

The average duration of prostheses in female patients was slightly higher (240.89 months) than in male patients (199.5 months), but this difference did not perform as statistically significant (MWU test, *p* = 0.119). The average duration of prostheses in younger and older patients was shown somewhat higher than in middle aged patients. Patients of the age of 46–50 years had the lowest prosthesis duration, but this difference did also not prove as statistically significant (Kruskal-Wallis test, *p* = 0.07). On the other hand, body mass (*Q*), daily exercise activity (*T*), and femoral offset (*h*) have all proven to have a statistically significant correlation (*p* < 0.05) with recorded duration *N*_*M*_, with exercise activity having the highest and most significant correlation *r* = −0.84, followed by body mass (*r* = −0.42) and femoral offset (*r* = −0.56). Therefore we can conclude that neither age nor gender has a significant impact on the expected duration of prostheses. Only body mass, exercise activity, and femoral offset have been shown statistically significant, and these are exactly the parameters that are involved in formula (18).

Moreover, we have performed a multivariate generalized regression analysis and compared it to our model based on the root mean square error (RMSE). The generalized regression model based on the same 15 patients resulted in the regression equation

**Figure 4:**
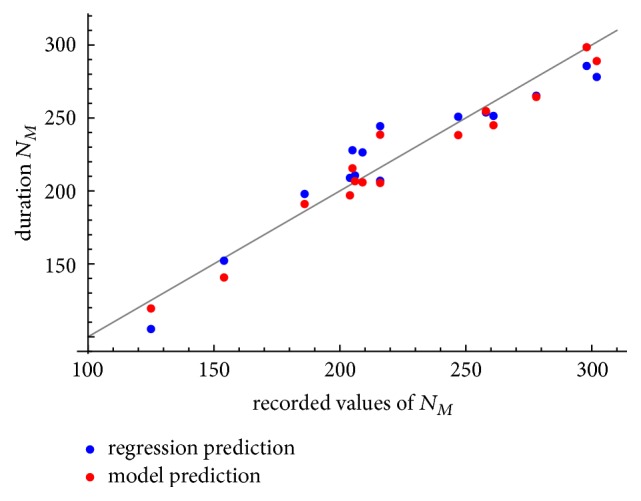
Comparison of the regression model (19) and the mathematical model (18).

(19) NM=−1.1834×Q−42.6465×T−2.2935×h−14.3738×K+633.0953

with a RMSE equal to 14.77 months. The RMSE for our nonlinear model given by (18) is equal to 10.39 months; thus our model clearly outperforms the regression model.  Figure 4 shows the scatterplot of the predicted values of durability *N*_*M*_ versus the observed values of *N*_*M*_, comparing the prediction of the linear regression with our model.

### 3.2. Model Interpretation and Deductions

Using the validated formulas (18) and (17), we can derive the effect of weight gain and weight loss, as well as the effect of increase or decrease of daily activity level on the expected life duration of a prosthesis, as given in Tables 2 and 3. For example, a patient weighing 60 kg can expect to increase the prosthesis duration for 8.7% after a weight loss of 10 kg. If the patient decreases his/her workout from 4 hours to 3 hours daily, the prosthesis will gain another 33.3% on its longevity.

From (17), we obtain an exact expression for predicting prostheses' durability *N*_*M*_ as a function depending on four variables: body weight *Q*, femoral offset *h*, daily physical activity *T*, and a weight factor *K*:

(20) $$\begin{array}{c} N_{M}=\frac{1}{T}{\left(\;\frac{2.61607405\times 10^{9}}{KQ\left(1+0.0555886592\times h\right)}\;\right)}^{1/2.187401}. \end{array}$$

**Figure 5:**
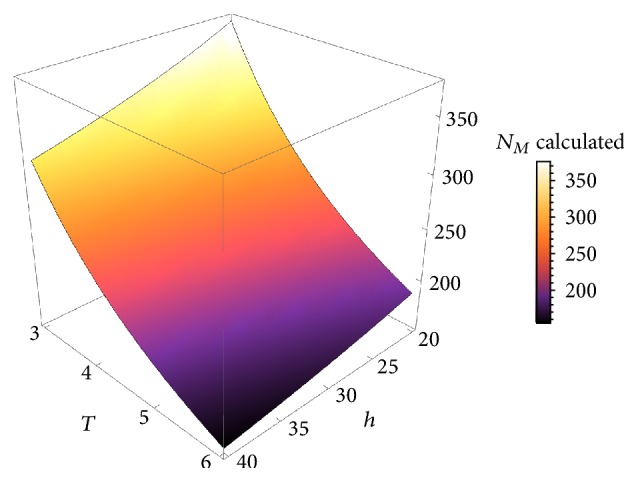
The shape of the function (20) for fixed values *h* = 30 and *K* = 4.

**Figure 6:**
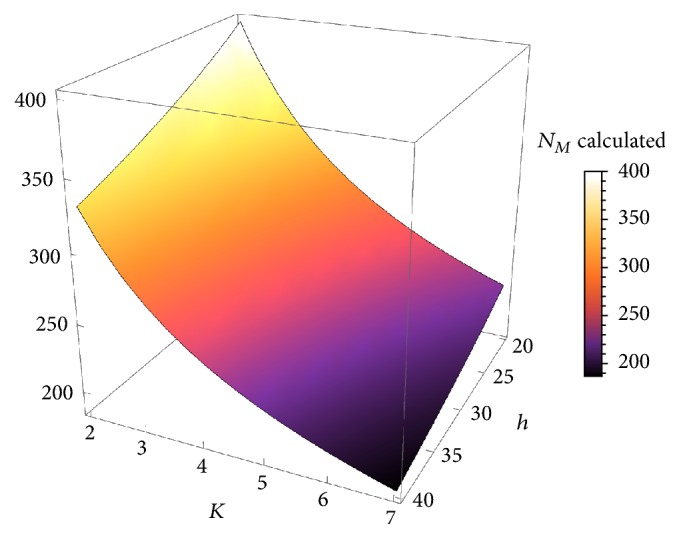
The shape of the function (20) for fixed values *Q* = 60 and *T* = 4.

**Figure 7:**
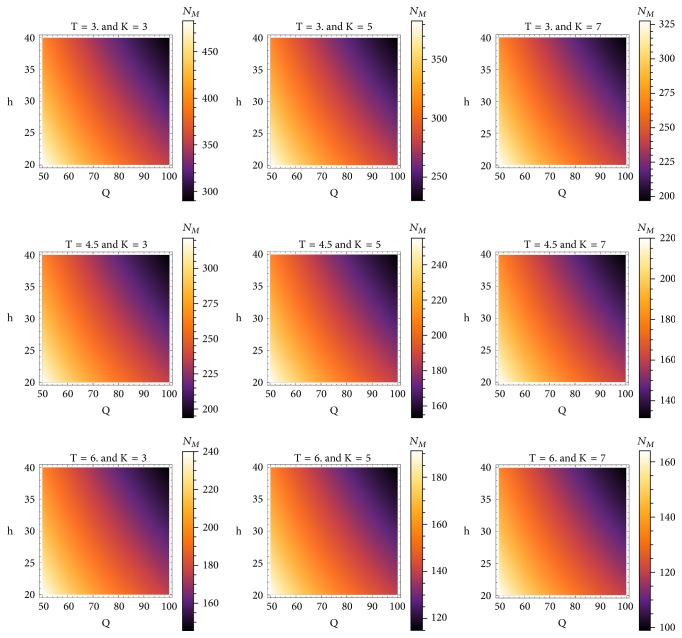
Contour plots of the function (20) for several values of *T* and *K*.

Figures 5, 6, and 7 depict the behavior of the function provided in (20). It can be seen how durability will decrease by increasing *T*, *K*, *Q*, or *h*.

On Figure 5, the independent variables are physical activity *T* ranging from 3 to 6 hours of daily exercise and body weight *Q* ranging from 50 to 100 kg. The other two variables are fixed at their mean values *h* = 30 and *K* = 4. Calculated durability of the prosthesis *N*_*M*_ varies from 140 months up to 360 months, with a steep descent. Similarly, Figure 6 shows the decay for fixed body weight *Q* = 60 and physical activity *T* = 4, as a function of femoral offset *h* ranging from 20 to 40 and weight factor *K* (involving age and behavior) ranging from 2 to 7.

On Figure 7, the independent variables are body weight *Q* ranging from 50 to 100 kg and femoral offset *h* ranging from 20 to 40 cm. The contour plots depict the variation of calculated durability *N*_*M*_ (darker shades correspond to lower durability and lighter shades correspond to higher durability) for several fixed choices of *T* and *K*. The impact of all four variables is highly significant and results in a decrease of the prosthesis durability.

### 3.3. Cross-Validation of the Model

In order to analyze the model error, we have performed a 10-fold cross-validation of the model. Triplets of three randomly chosen patients were formed during ten runs of the test, each time calculating the parameters *c*_1_, *c*_2_, and *c*_3_ based on the three chosen patients, then evaluating the model prediction *N*_*M*_ = (1/*T*)(*c*_2_/*KQ*(1+*c*_1_×*h*))^−1/*c*_3_^, and finally testing the model on the remaining fifteen patients.  Table 4 summarizes quantitatively the performance of our model in terms of MSE, RMSE, MBE, MAE, R, FACT2, and IA for each test run, while Table 5 shows the mean and standard error of each statistical metrics.  Table 6 summarizes the mean value and standard deviation of the calculated parameters *c*_1_, *c*_2_, and *c*_3_, as well as their 95% confidence interval.

Statistical metrics used to measure the predictive performance of the model:

1. Mean Squared Error (MSE): MSE = (1/15)∑_*i*=1_^15^(*N*_*M*,*i*_ − *N*_*i*_)^2^
2. Root Mean Squared Error (RMSE): $RMSE=\sqrt{MSE}$
3. Relative Root Mean Squared Error: $relativeRMSE=100\left(RMSE/\bar{N}\right)$
4. Mean Absolute Error (MAE): MAE = (1/15)∑_*i*=1_^15^|*N*_*M*,*i*_ − *N*_*i*_|
5. Mean Bias Error (MBE): $MBE=\left(1/15\right)\sum_{i=1}^{15}(N_{M,i}-N_{i})=\bar{N_{M}}-\bar{N}$
6. Mean Absolute Percentage Error (MAPE): $MAPE=100\left(MAE/\bar{N}\right)$
7. Correlation coefficient (*R*): $R=\left(1/15\right)\sum_{i=1}^{15}N_{M,i}\times N_{i}-\bar{N_{M}}\times \bar{N}/\sigma (N_{M})\sigma (N)$
8. Standard Deviation of Residuals (SDR): $SDR=\sqrt{RMSE^{2}-MBE^{2}}$
9. Fraction of prediction within a Factor of Two (FACT2): $FACT2=\left(1/14\right)\sum_{i=1}^{15}\;\left(\left(\left(N_{M,i}-\bar{N_{M}}\right)/\sigma \left(N_{M}\right)\right)\left(\left(N_{i}-\bar{N}\right)/\sigma (N)\right)\right)$, that is, the fraction of model predictions that satisfy 1/2 ≤ *N*_*M*,*i*_/*N*_*i*_ ≤ 2
10. Index of Agreement (IA): $IA=1-\sum_{i=1}^{15}(N_{M,i}-N_{M}{)}^{2}/\sum_{i=1}^{15}(|N_{M,i}-\bar{N_{M}}|+|N_{i}-\bar{N}|{)}^{2}$

Based on all values of error indices assessing the model's predictive performance, we may conclude that the model performs well (e.g., average RMSE is 13.5 months with a 95% confidence interval ranging from 11 months up to 16 months). Extremely high values of the correlation coefficient (average *R* is 0.9765 with a very small standard deviation of 0.00646) show that the predicted values highly correlate with the recorded values. A positive average MBE indicates that our model tends to slightly overestimate the prosthesis duration rather than underestimating it.

## 4. Discussion

Aseptic loosening is commonly seen as an increased width of radiolucent lines about the prosthesis on radiographs and is usually associated with pain. Heavy repetitive impacts associated with running, jumping, and high-level sport activities can increase the risk of progressive aseptic loosening of a prosthesis [21]. Indeed, our analysis reveals that weight and activity levels can highly contribute to the development and progression of aseptic loosening. Our mathematical model has shown that extreme physical activity (jumping, stumbling without falling, heavy physical work, extreme sports activity, etc.) even for young patients substantially affects prosthetic joint longevity. It was also shown in [22] that the physical activity of a patient (such as sports activity) that includes impact loading influences the loosening of prostheses.

However, we strongly encourage and promote moderate physical activity as it shows that patients activity correlates with better and faster bone-stem incorporation. A key innovation of this study is the development of a mathematical model for the durability of hip prostheses. In this study we prefer to use a mathematical model as a starting point. Our investigations are based on a mechanical model with a force action on the hip consisting of three components as it is explained in Section 3. Two of them, producing the torsion, were sometimes neglected. However, it is known that torsion effects the aseptic loosening. Since the experimental results concerning the effects of torsion were not quite clearly involved in any existing mathematical model, we included torsion effects through the coefficient *K*, determined by the lifestyle analysis of each patient. This aspect of our model needs improvements planned in our further investigations. The lifestyle of a patient must be incorporated through certain factors in a formula that predicts the durability of his/her prosthesis.

The maximal stress about prostheses is founded on the work of Da Silva [16] who discussed and implemented the theory of eccentric compression (bending superposed on compression and torsion). Existing mathematical models are based on heat transfer, fluid flow, and stress distribution usually related to cemented hip replacement [11]. However, experimental confirmations of such models are not given. In our approach, we are following a universal model related to the fatigue of heterogeneous materials [2]. Our mathematical model is tested for prostheses of well-operated patients, with several simplifications, in order to show the validity of the assumed mechanical principles. The experimental data are analyzed showing clearly preferences of our prediction formula for durability given at the end of the paper. Tsai et al. in [19] used simulations of real conditions to determine fatigue. Ploeg et al. in [12] used mechanical testing to estimate failure of prostheses.

We note that durability of femoral prostheses was studied in [23] on 95 patients with osteonecrosis, without developing a mathematical model. Mallory showed [24] that the femoral component geometry influences the durability of a prosthesis, while in [25] it was shown that even engravings may be the reason for mechanical failure of a prosthesis.

Based on the fact stated in [10] that loosening is primarily a mechanical phenomenon, we analyzed the mechanical phenomenon leading to aseptic loosening of the hip prosthesis.

We derived our formula based on results presented in [3] with modification, concerning stresses, which are specific to the fatigue life of a hip prosthesis. The coefficients in the formula can be calculated from a randomized sample of patients with the same prosthesis type and then validated by assessing predicted and actual outcomes in a larger group of patients with the same prosthesis type. The central results of our study are presented in Tables 1, 2, and 3 and formula (18). Ideally, patient specific data such as body mass index and activity levels would be readily available and not require additional advanced testing.

The proposed model is based on engineering formulas (Coffin/Manson equation and Basquin's law) regarding heterogeneous materials. The central assumption is that the system hip implant, femur, represents a heterogeneous system, as stated in [2]. Based on this, we proposed a formula for the durability (fatigue life) of a hip prosthesis. The specific feature of (17) is that the stress is calculated by the use of the formula for eccentric compression. This involves the introduction of a value *h* (femoral offset) which is specific for each patient. Values for the daily activity *T* are estimated after the interview with each patient where his/her lifestyle and professional occupation are analyzed. The coefficient *K* is also estimated from the patient's mobility and excess of physical activities. In estimating *K* we followed the results of [26] and [11]; see (7). Those authors studied the effective force (in our notation *KQ*) in detail. They showed that there exist two maxima of a force value during a single step and that the greater maxima determine *K*. The influence of footwear on the effective force *KQ* was studied by Sinclair [27]. This effect can be in a further study included in our formula. The influence of the different femoral ball sizes on the stress and deformation and consequently on the durability of implant was studied in [28]. In our study, the femoral ball sizes were constant for each patient so this effect did not influence our results. Dynamic load during walking, as a function of time, is also a key stressor [11]. This factor is also included in our formula through *K*.

For the purposes of defining the coefficients unique to this prosthesis, we randomly chose three patients. This allowed us to introduce their data into the equation to determine the coefficients. The subsequent formula given in (20) with prosthesis specific coefficients was then applied to the remaining 15 patients to come up with a predicted durability for that patient.

The agreement between predicted and observed fatigue life *N*_*M*_ is quite positive. Predicted and observed values were compared using the Mann–Whitney *U* test of ranks resulting in a high *p* value (*p* = 0.709) that indicates concurrence of the calculated and recorded values of *N*_*M*_. This confirms validity of the proposed mathematical model. Moreover, this model outperforms by its RMSE even the regression models.

The full model for exact expression for durability predicting, a formula of the type obtained earlier in [15], in our case is (20), where *K* is given by (15). As could be seen the load coefficient *K* depends on force coefficient *k*_1_ and torsion coefficient *k*_2_.

Our derived mathematical model focuses on predicting prosthesis failure due to aseptic loosening even that we know this is not main reason of vast majority of prosthesis failures. We are very familiar that there are patients out there with similar age, body weight, and activity level that did not undergo THA revision due to nonfailure of hip prosthesis. The model we developed serves to forecast prosthesis behavior and duration only due to aseptic loosening but it does not describe failure due to other reasons, and neither does it forecast duration in those patients who will never experience failure. A complete probabilistic model for overall duration may be developed using Bayesian techniques by classifying patients into different cohorts each corresponding to a different cause of prosthesis failure. Developing a separate model for each failure cause, then assessing the individual predicted durations, and weighing them over all cohorts would provide a better and more general way of prediction for overall duration of a prosthesis.

Limitations of the current study are a retrospective design, only one type of a hip implant, relatively small sample size, and not the most accurate questionnaire on lifestyle and habits of the patients. We must point out that we chose patients with aseptic loosening only due to fatigue. Our study was fully based on HA coated stems as those were the ones mostly used in our hospital at that time. It is our opinion that our derived formula would also be widely applicable at other types of stem coatings (plasma spray, porous, grit blasted, etc.) as well by changing coefficients within mathematical model.

Another limitation factor of our study affecting the predictive ability for a general population could be the fact that we have chosen exclusively young and active patients with good bone quality and without diagnosed or even assumed osteoporosis. Also, regarding demographic factors we totally disregarded patients education level, income level, religion, occupation, and marital status and included only sex and age. Another very important limitation of our study relates to biological factors. It is well known that particulate debris produced by implants is a major factor of aseptic loosening-particulate disease. However, we found no substantial particulate debris during THA revision surgeries for group of patients included in this study.

Analyzing the overall performance of the model we have found no evidence that would indicate that the model might be improved by “tailoring” the coefficients to a specific patient cohort. For example, one might expect that choosing three elderly female patients to evaluate *c*_1_, *c*_2_, and *c*_3_ would provide a model formula that better predicts the prosthesis duration to other elderly female patients than to young male patients or to a mixed group of patients. Interestingly, this is not the case, and the model preforms in an equal manner no matter how the three patients are chosen. However, we emphasize that our formula works best for the values of *K* between 2.2 and 7.2, which is a consequence of the fact that we used interpolation between these values (Figure 2), and other extrapolated values are less confident.

Also, formula (20) loses on its performance for values of *T* that are very close to zero, which is a mathematical consequence (predicted values would tend to be infinite). Therefore, we do not analyze the case when patients are not physically active at all (e.g., patients bound to bed) and neither do we promote a complete absence of physical activity. Our model describes the contribution of physical activity from a reasonable moderate to a high range (best from 2 to 7 hours of daily physical activity) to prosthesis duration.

It must be emphasized that the formula with coefficients applied to the 15 patients in the study group is prosthesis specific and that further study is needed to estimate the parameters of (18) for other hip prostheses (other materials by different manufacturers), especially to test materials proposed in [4, 29]. Nonetheless, we are confident that these coefficients can be easily and efficiently determined by following the method outlined above. The Questionnaire related to coefficients *K* and *T* may also be improved in the future in order to minimize subjective components of the input regarding examinee related to a level of activity, lifestyle, and the time spent “on foot.” We would predict that, with improved lifestyle analysis, our proposed mechanical model could give even better results. To our knowledge, there is no questionnaire that can precisely determine the factors *k*_1_ and *k*_2_ (activity and lifestyle habits) in our formula.

## 5. Conclusion

This study demonstrates the fact that, with the application of a mathematical model and the input of individualized patient information, one can confidently predict the durability of a prosthesis for an individual patient with a standard error of ca. 10 months. We believe these are important pieces of information for several reasons:

- The model follows the principle of parsimony: we are able to calculate the coefficients of the model based on only three patients. The precision of statistical models such as regression models depends on the sample size and one requires a large number of patients to obtain accuracy of the estimated parameters. Our model achieves a higher precision and better prediction based on the fixed number of three patients.
- The model can help the manufacturer of the prosthesis to calculate its warranty and to provide an insight into further improvements depending on the material characteristics.
- Once the femoral offset *h* is measured from the X-ray diagnostics, the orthopedist may accurately estimate the prosthesis durability for the specific patient based on the patient's lifestyle (physical activity) and body weight.

## Figures and Tables

**Table 1:** Calculated and recorded life of a hip prosthesis.

Patient	Age	Gender	Body mass *Q* (kg)	Activity *T* (hours per day)	Length *h* (mm)	Normalized score of physical activity (0-1)	Weight coefficient *K*	*N*_*M*_ calculated (months)	*N*_*M*_ recorded (months)
1	40	F	50	4	25.6	0.537	4.04	297.2	298
2	47	F	65	5	36.7	0.545	4.08	189.2	186
3	53	M	80	4	31.2	0.703	5.00	205.8	209
4	55	F	65	5	24	0.537	4.04	214.5	205
5	39	M	78	3.5	31.1	0.702	5.00	238.1	247
6	42	F	57	4.5	30.3	0.571	4.22	232.7	216
7	41	M	80	4	26.1	0.414	3.44	256.6	258
8	42	F	50	5.5	31.2	0.532	4.01	205.2	216
9	54	F	70	4	30	0.521	3.96	246.1	261
10	48	M	100	6	35.4	0.712	5.06	118.7	125
11	44	F	55	5	36.4	0.543	4.07	205	206
12	50	M	70	6	26.4	0.841	5.95	141.2	154
13	42	M	70	5	29.5	0.542	4.07	195.4	204
14	36	F	60	4	29.4	0.551	4.11	261.1	278
15	54	F	50	4	28.8	0.545	4.08	286.4	302
16^*∗*^	37	F	58	4	25.2	0.702	5.00	253	253
17^*∗*^	41	F	65	4	32.4	0.975	6.99	192	192
18^*∗*^	40	F	45	4	34.4	0.529	4.00	288	288

**Table 2:** Influence of the body mass change on the prosthesis life.

Current body mass (kg)	50	60	70	80	90	100
Expected increase in prosthesis life duration after a weight loss of 10 kg	10.7%	8.7%	7.3%	6.3%	5.5%	4.9%
Expected decrease in prosthesis life duration after a weight gain of 10 kg	8%	6.8%	5.9%	5.2%	4.7%	4.2%

**Table 3:** Influence of the activity (hours/day) change on the prosthesis life.

Current workout activity (hours/day)	3	3.5	4	4.5	5	5.5	6
Expected increase in prosthesis life duration after decrease of exercise 1 hour per day	50%	40%	33.3%	28.6%	25%	22.2%	20%
Expected decrease in prosthesis life duration after an increase of exercise 1 hour per day	25%	22.2%	20%	18.2%	16.7%	15.4%	14.3%

**Table 4:** Model evaluation parameters for each of the ten test runs in the cross-validation.

Run	1	2	3	4	5	6	7	8	9	10
Random chosen patients	3, 9, 17	1, 5, 18	12, 14, 15	2, 7, 11	1, 5, 10	4, 11, 17	1, 7, 8	5, 11, 18	1, 2, 16	3, 13, 16
*c*_1_	0.0381874	0.069505	0.06584	0.055514	0.050804	0.018742	0.002836	0.196751	0.084587	0.001015
*c*_2_	1.10642E + 8	1.9208E + 10	2.16E + 10	9.83508E + 7	1.1614E + 10	2.36563E + 9	9.20321E + 8	2.8813E + 10	2.50708E + 09	4.21472E + 7
*c*_3_	−1.74564	−2.44913	−2.45388	−1.71357	−2.40479	−2.25638	−2.1546	−2.38906	−2.1433	−1.71418
MSE	343.098	93.1858	256.19	198.717	91.3597	180.136	169.342	103.721	131.056	361.857
RMSE	18.5229	9.65328	16.0059	14.0967	9.55823	13.4215	13.0132	10.1844	11.448	19.0225
Relative RMSE (%)	8.08625	4.43489	7.13702	6.13256	4.18242	5.7603	5.86883	4.55066	5.10919	8.31405
MAE	15.5688	8.16701	13.987	12.8838	8.25818	11.8901	10.1982	8.80112	10.1294	13.9091
MBE	12.4579	0.20466	13.987	−5.31764	0.63503	−9.551	5.66352	2.31164	−6.75237	10.1132
MAPE (%)	6.79663	3.75207	6.23679	5.60488	3.61355	5.10303	4.59932	3.93258	4.52073	6.07914
R	0.979059	0.977203	0.984075	0.97586	0.972876	0.982913	0.970239	0.979459	0.980572	0.962632
SDR	13.7076	9.65111	7.78158	13.0553	9.53711	9.42946	11.7161	9.91855	9.24456	16.1115
FACT2	0.979059	0.977203	0.984075	0.97586	0.972876	0.982913	0.970239	0.979459	0.980572	0.962632
IA	0.970791	0.987729	0.965396	0.982194	0.985253	0.981262	0.981396	0.989074	0.984831	0.968858

**Table 5:** Model prediction error analysis based on a 10-fold cross-validation.

	MSE	RMSE	Relative RMSE	MAE	MBE	MAPE	R	SDR	FACT2	IA
Mean	192.8663	13.4927	5.9576	11.3793	2.3752	5.0239	0.9765	11.0153	0.9765	0.9797
Standard deviation	98.82318	3.46642	1.4803	2.64451	8.16261	1.12062	0.00646	2.56534	0.00646	0.00832

**Table 6:** Model parameter error analysis based on a 10-fold cross-validation.

Model parameter	Mean value	Standard deviation	Confidence −95%	Confidence +95%
*c*_1_	0.058378	0.056207	0.01817	0.098586
*c*_2_	8.728087E + 09	1.080951E + 10	9.9543054E + 08	1.646074E + 10
*c*_3_	−2.142453	0.308872	−2.363407	−1.921499

## Appendix

### Questionnaire on Individual Patient's Physical Activities

*Questionnaire on Physical Activities and Lifestyle Habits*

On average, how many hours daily were you physically active during the past year? —

*Under each heading, please tick the ONE box that best describes your mobility activities during the past YEAR.*

Regular daily activities (e.g., walking, climbing stairs, carrying weight after shopping etc.)

  I am extremely engaged in mobility activities □
  I am much engaged in mobility activities □
  I am moderately engaged in mobility activities □
  I am little engaged in mobility activities □
  I am insignificantly engaged in mobility activities □

Household activities (e.g., sitting down, standing up, standing up from bed, etc.)

  I am extremely engaged in mobility activities □
  I am much engaged in mobility activities □
  I am moderately engaged in mobility activities □
  I am little engaged in mobility activities □
  I am insignificantly engaged in mobility activities □

Work related activities (e.g., walking, carrying weight, etc.)

  I am extremely engaged in mobility activities □
  I am much engaged in mobility activities □
  I am moderately engaged in mobility activities □
  I am little engaged in mobility activities □
  I am insignificantly engaged in mobility activities □

Excessive activities (e.g., running, jogging, jumping, playing sports, etc.)

  I am extremely engaged in mobility activities □
  I am much engaged in mobility activities □
  I am moderately engaged in mobility activities □
  I am little engaged in mobility activities □
  I am insignificantly engaged in mobility activities □

*We would like to know your OVERALL intensity of physical activity during the past year.*

- This scale is numbered from 0 to 100.
  - 100 means you are extremely highly involved in high-impact physical activities.
  - 0 means you are very little involved in physical activities.
- Mark an X on the scale to indicate your average amount of physical activities during the past YEAR.

Now, please write the number you marked on the scale in the box below.

YOUR AMOUNT OF MOBILITY ACTIVITIES = *▭*

## References

[1] Runciman A., Xu D., Pelton A. R., Ritchie R. O. (2011). An equivalent strain/Coffin-Manson approach to multiaxial fatigue and life prediction in superelastic Nitinol medical devices. *Biomaterials*.

[2] Kun F., Carmona H. A., Andrade J. S., Herrmann H. J. (2008). Universality behind basquin’s law of fatigue. *Physical Review Letters*.

[3] Meyers M. A., Chawla K. K. (2008). *Mechanical Behavior of Materials*.

[4] Semlitsch M., Willert H. G. (1980). Properties of implant alloys for artificial hip joints. *Medical & Biological Engineering & Computing*.

[5] Klippel J. H., Stone J. H., Crofford L. J., White P. H. (2008). Primer on the rheumatic diseases: Thirteenth edition. *Primer on the Rheumatic Diseases: Thirteenth Edition*.

[6] Callaghan J. J. (2015). *The Adult Hip: Hip Arthroplasty Surgery*.

[7] Schwartz B. E., Piponov H. I., Helder C. W., Mayers W. F., Gonzalez M. H. (2016). Revision total hip arthroplasty in the United States: national trends and in-hospital outcomes. *International Orthopaedics*.

[8] Kurtz S., Mowat F., Ong K., Chan N., Lau E., Halpern M. (2005). Prevalence of primary and revision total hip and knee arthroplasty in the United States from 1990 through 2002. *The Journal of Bone and Joint Surgery—Series A*.

[9] Siopack J. S., Jergesen H. E. (1995). Total hip arthroplasty. *Western Journal of Medicine*.

[10] Bono J. V., Scott R. D. (2005). Revision total knee arthroplasty. *Revision Total Knee Arthroplasty*.

[11] Srimongkol S. (2012). Mathematical modeling for stress distribution in total hip arthroplasty. *International Journal of Mathematical Models and Methods in Applied Sciences*.

[12] Ploeg H.-L., Bürgi M., Wyss U. P. (2009). Hip stem fatigue test prediction. *International Journal of Fatigue*.

[13] Radonovich D., Gordon A. P. Methods of extrapolating low cycle fatigue data to high stress amplitudes.

[14] Stojanac I., Drobac M., Petrovic L., Atanackovic T. (2012). Predicting in vivo failure of rotary nickel-titanium endodontic instruments under cyclic fatigue. *Dental Materials*.

[15] Moore T. L. A., O'Brien F. J., Gibson L. J. (2004). Creep does not contribute to fatigue in bovine trabecular bone. *Journal of Biomechanical Engineering*.

[16] Da Silva V. D. (2006). Mechanics and strength of materials. *Mechanics and Strength of Materials*.

[17] Bergmann G., Graichen F., Rohlmann A. (2004). Hip joint contact forces during stumbling. *Langenbeck's Archives of Surgery*.

[18] Varady P. A., Glitsch U., Augat P. (2015). Loads in the hip joint during physically demanding occupational tasks: A motion analysis study. *Journal of Biomechanics*.

[19] Tsai A. G., Reich M. S., Bensusan J., Ashworth T., Marcus R. E., Akkus O. (2013). A fatigue loading model for investigation of iatrogenic subtrochanteric fractures of the femur. *Clinical Biomechanics*.

[20] Bergmann G., Deuretzbacher G., Heller M. (2001). Hip contact forces and gait patterns from routine activities. *Journal of Biomechanics*.

[21] Röder C., Eggli S., Münger P., Melloh M., Busato A. (2008). Patient characteristics differently affect early cup and stem loosening in THA: a case-control study on 7,535 patients. *International Orthopaedics*.

[22] Kilgus D. J., Dorey F. J., Finerman G. A. M., Amstutz H. C. (1991). Patient activity, sports participation, and impact loading on the durability of cemented total hip replacements. *Clinical Orthopaedics and Related Research*.

[23] Han S.-I., Lee J.-H., Kim J. W., Oh C. W., Kim S.-Y. (2013). Long-term durability of the CLS femoral prosthesis in patients with osteonecrosis of the femoral head. *The Journal of Arthroplasty*.

[24] Mallory T. H. (1987). Femoral component geometry. A factor in total hip arthroplasty durability. *Clinical Orthopaedics and Related Research*.

[25] Kluess D., Steinhauser E., Joseph M. (2015). Laser engravings as reason for mechanical failure of titanium-alloyed total hip stems. *Archives of Orthopaedic and Trauma Surgery*.

[26] Zadpoor A. A., Nikooyan A. A. (2011). The relationship between lower-extremity stress fractures and the ground reaction force: a systematic review. *Clinical Biomechanics*.

[27] Sinclair J. (2014). Effects of barefoot and barefoot inspired footwear on knee and ankle loading during running. *Clinical Biomechanics*.

[28] Shaik S. A., Bose K., Cherukuri H. P. (2012). A study of durability of hip implants. *Materials and Corrosion*.

[29] Ikeda D., Saito M., Murakami A., Shibuya T., Hino K., Nakashima T. (2000). Mechanical evaluation of a bio-active bone cement for total hip arthroplasty. *Medical & Biological Engineering & Computing*.

